# Supplementary material for: Cancer-specific association between Tau (MAPT) and cellular pathways, clinical outcome, and drug response
Source: Sci Data. 2023 Sep 20;10:637. doi: 10.1038/s41597-023-02543-y (PMC10511431; doi:10.1038/s41597-023-02543-y)
Supplement: Supplementary file 1 — Supplementary Figures and Tables [file 41597_2023_2543_MOESM1_ESM.pdf]

Supplementary Figure 1

a

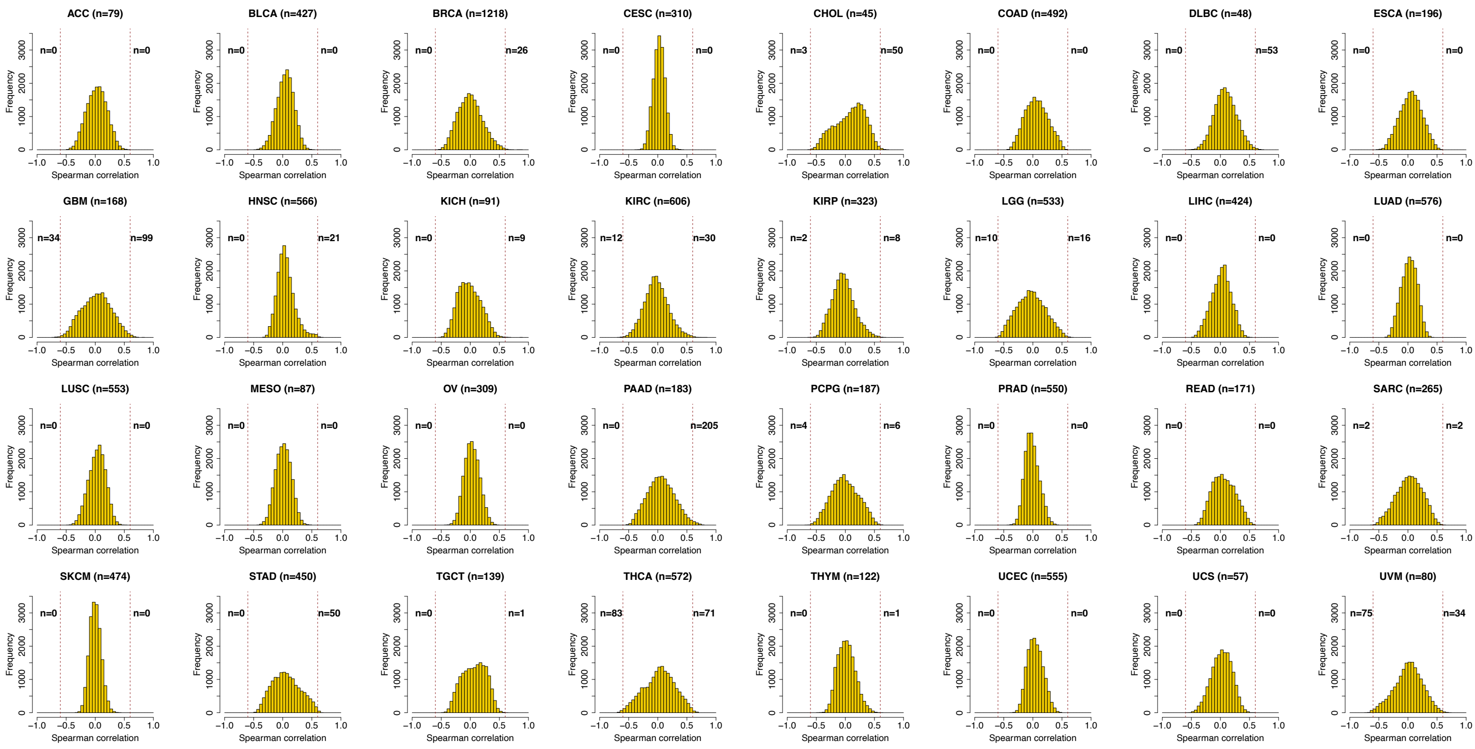

b

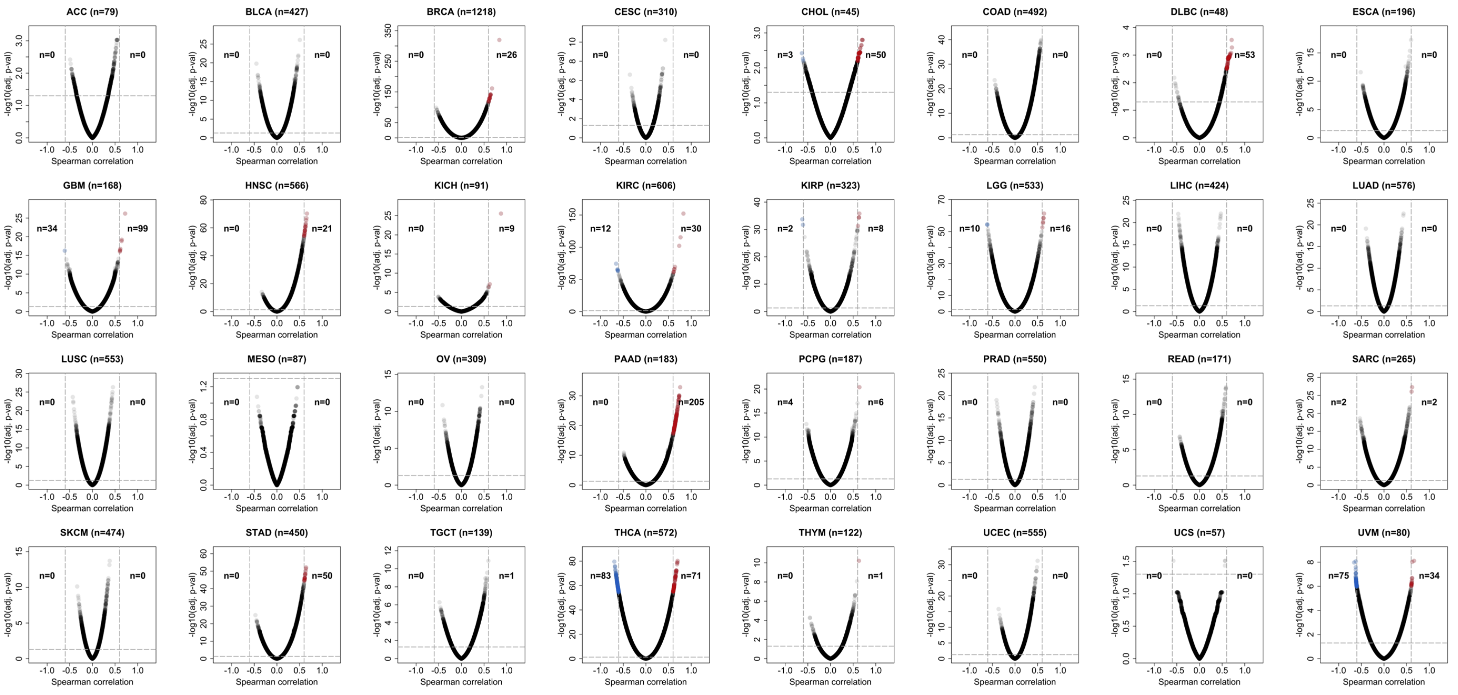

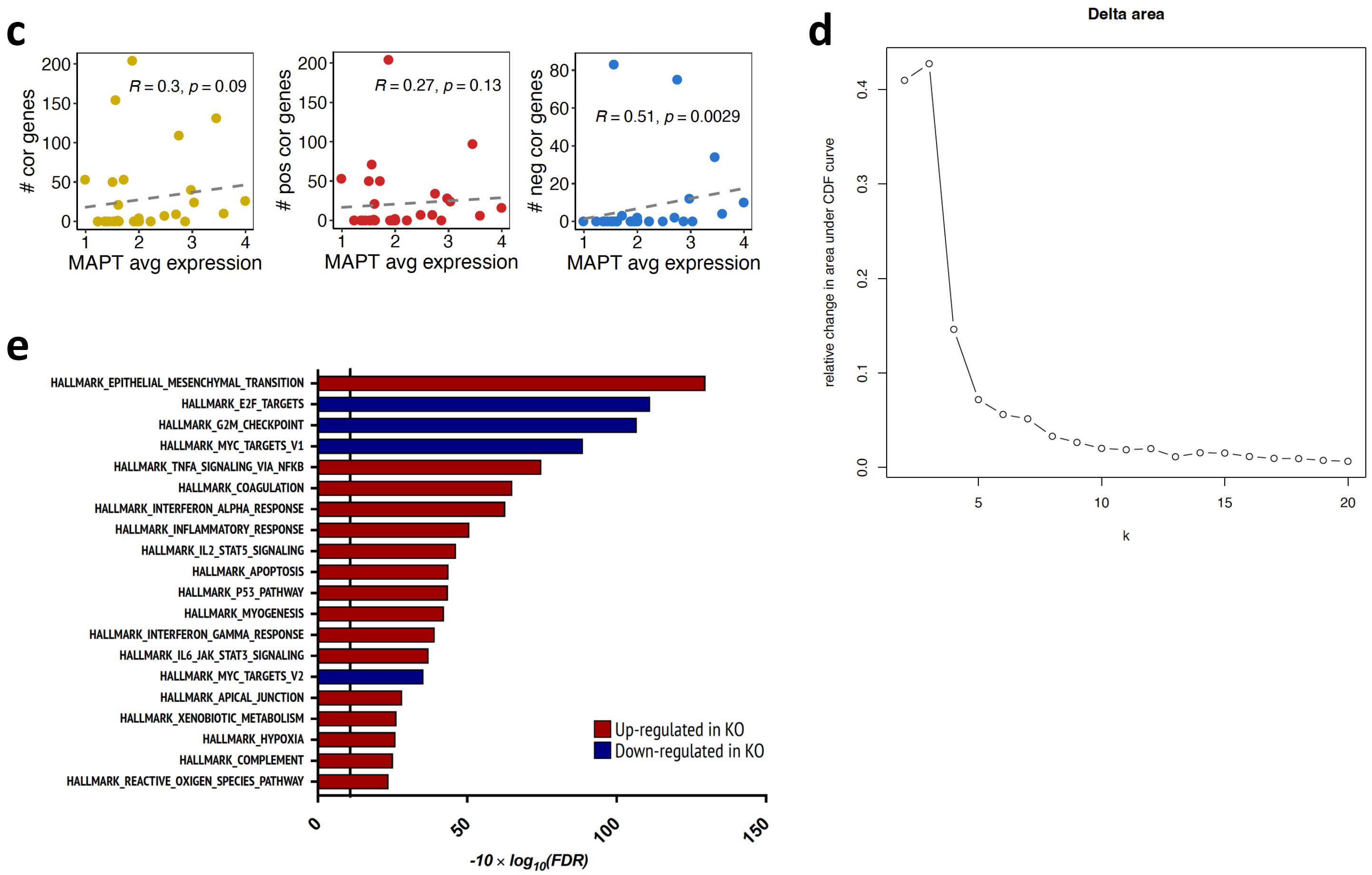
c
R = 0.3, p = 0.09
# cor genes
MAPT avg expression
R = 0.27, p = 0.13
# pos cor genes
MAPT avg expression
R = 0.51, p = 0.0029
# neg cor genes
MAPT avg expression
d
Delta area
relative change in area under CDF curve
k
e
HALLMARK_EPITHELIAL_MESENCHYMAL_TRANSITION
HALLMARK_E2F_TARGETS
HALLMARK_G2M_CHECKPOINT
HALLMARK_MYC_TARGETS_V1
HALLMARK_TNFA_SIGNALING_VIA_NFKB
HALLMARK_COAGULATION
HALLMARK_INTERFERON_ALPHA_RESPONSE
HALLMARK_INFLAMMATORY_RESPONSE
HALLMARK_IL2_STAT5_SIGNALING
HALLMARK_APOPTOSIS
HALLMARK_P53_PATHWAY
HALLMARK_MYOGENESIS
HALLMARK_INTERFERON_GAMMA_RESPONSE
HALLMARK_IL6_JAK_STAT3_SIGNALING
HALLMARK_MYC_TARGETS_V2
HALLMARK_APICAL_JUNCTION
HALLMARK_XENOBIOTIC_METABOLISM
HALLMARK_HYPOXIA
HALLMARK_COMPLEMENT
HALLMARK_REACTIVE_OXIGEN_SPECIES_PATHWAY
Up-regulated in KO
Down-regulated in KO
-10 × log10(FDR)

Supplementary figure 2

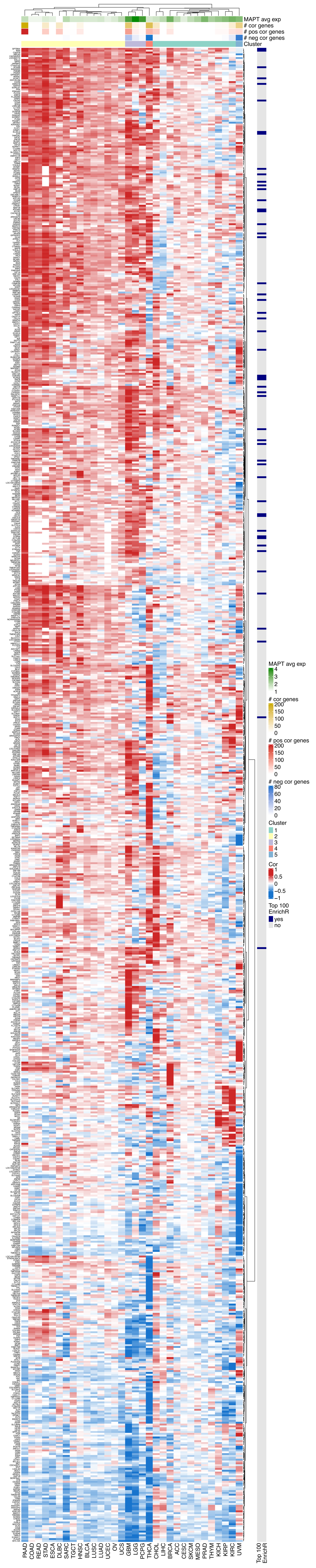

Supplementary Figure 3

a

Integrin signalling pathway

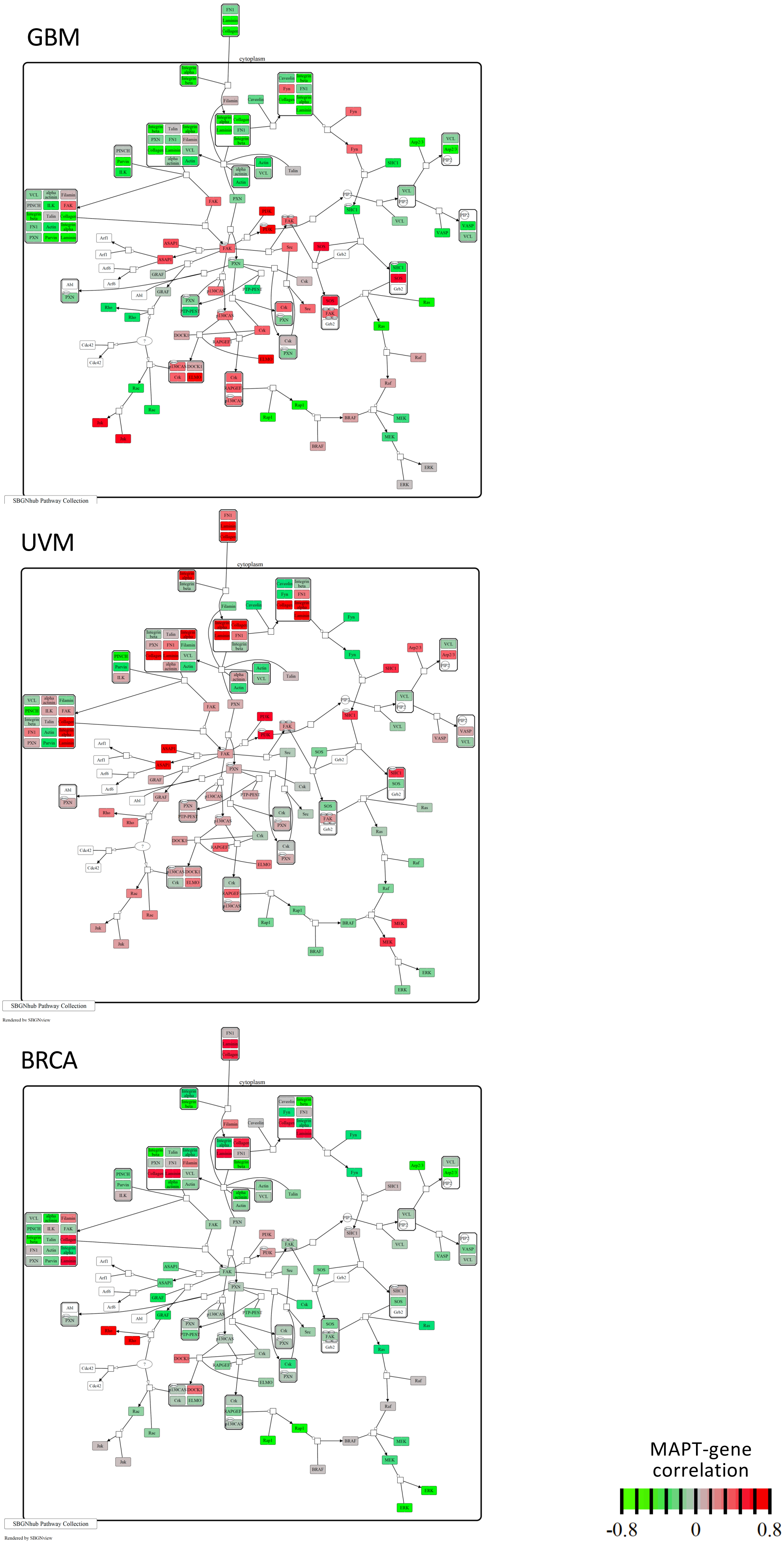

b

DNA replication

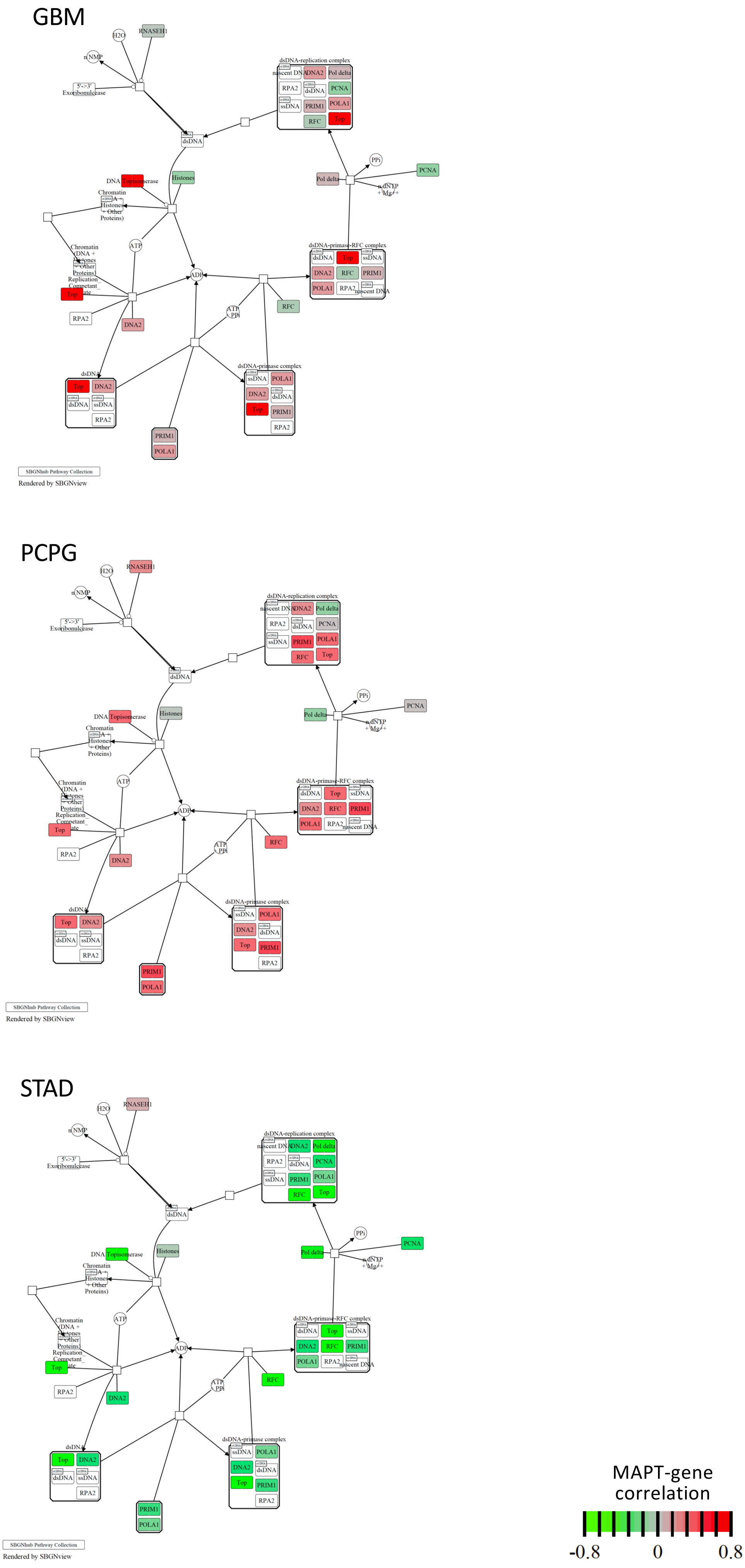

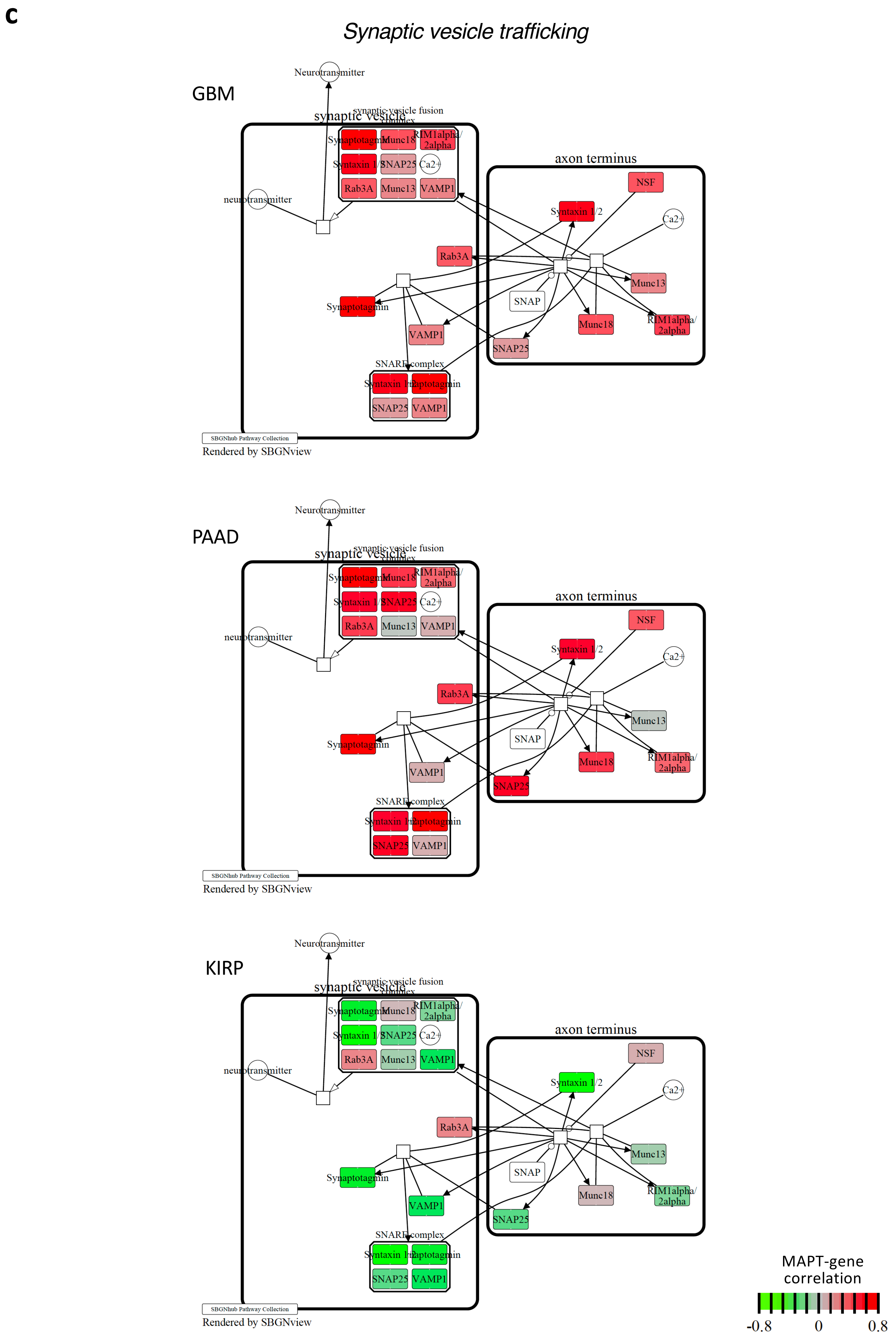
c
Synaptic vesicle trafficking
GBM
PAAD
KIRP
Neurotransmitter
neurotransmitter
synaptic vesicle
synaptic vesicle fusion complex
Synaptotagmin
Munc18
RIM1alpha/2alpha
Syntaxin 1/2
SNAP25
Ca2+
Rab3A
Munc13
VAMP1
axon terminus
NSF
SNAP
SNARE complex
SBGNhub Pathway Collection
Rendered by SBGNview
MAPT-gene correlation
-0.8
0
0.8

Supplementary Figure 4

a

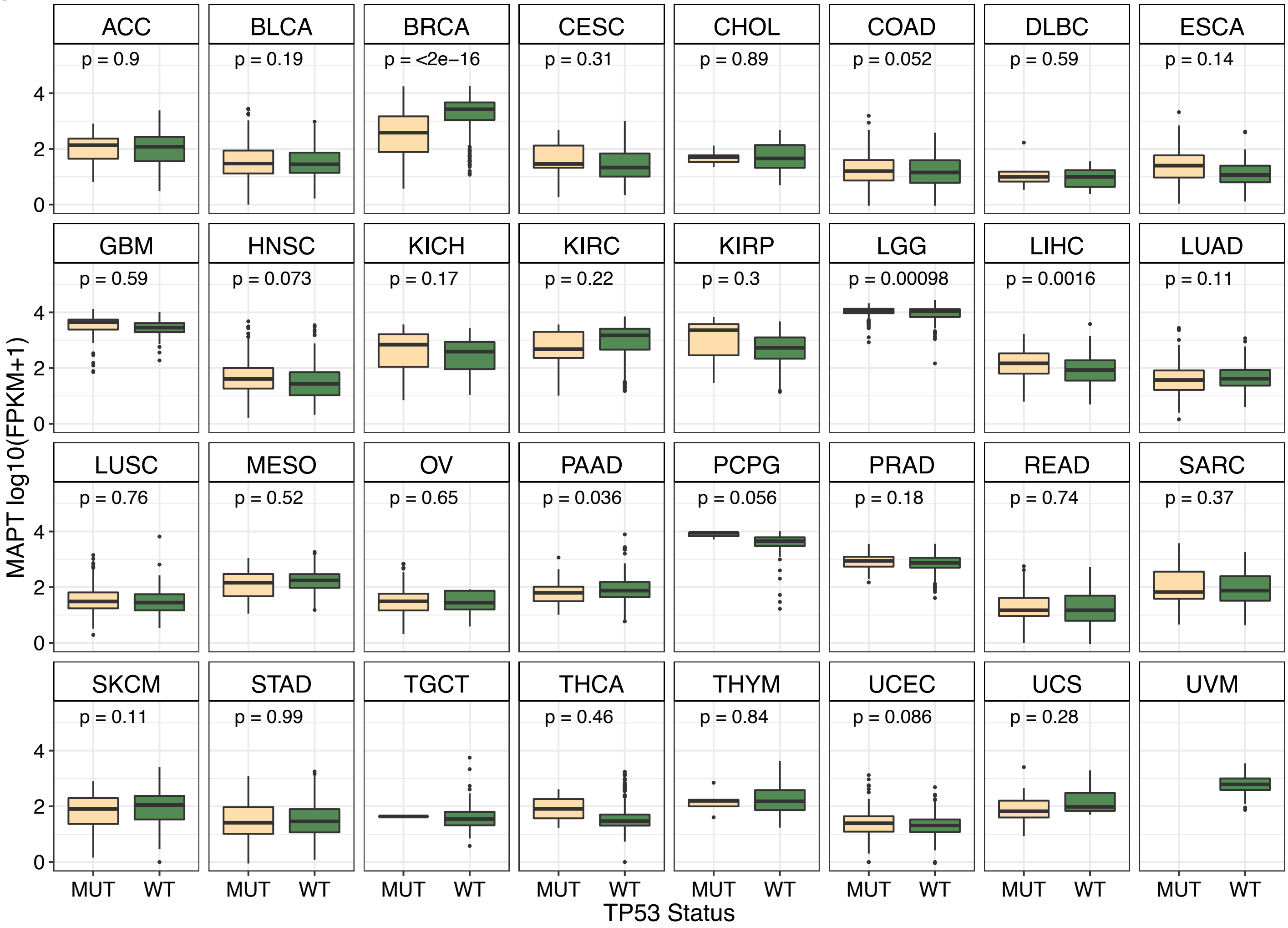

b

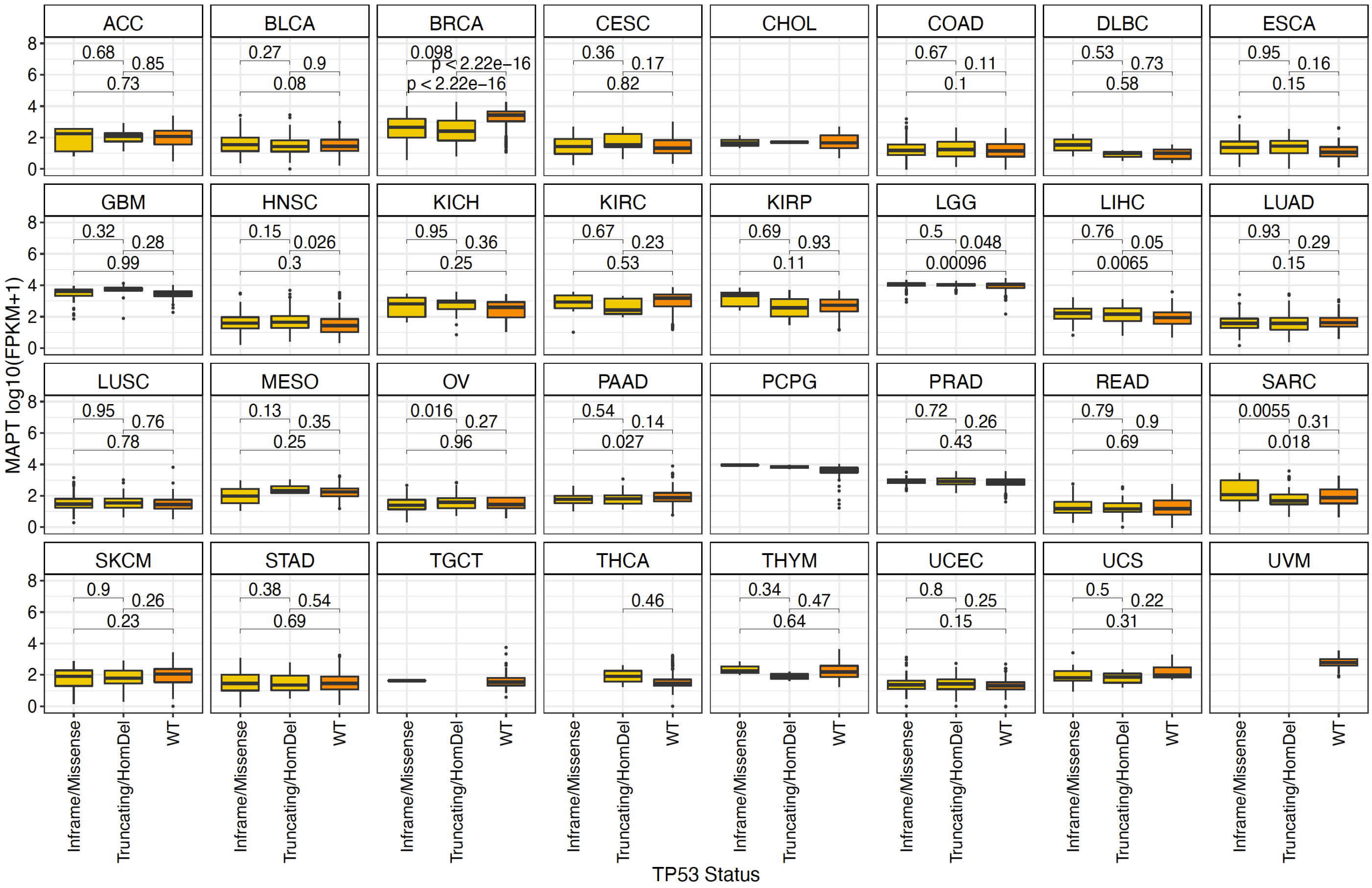

Supplementary Figure 5

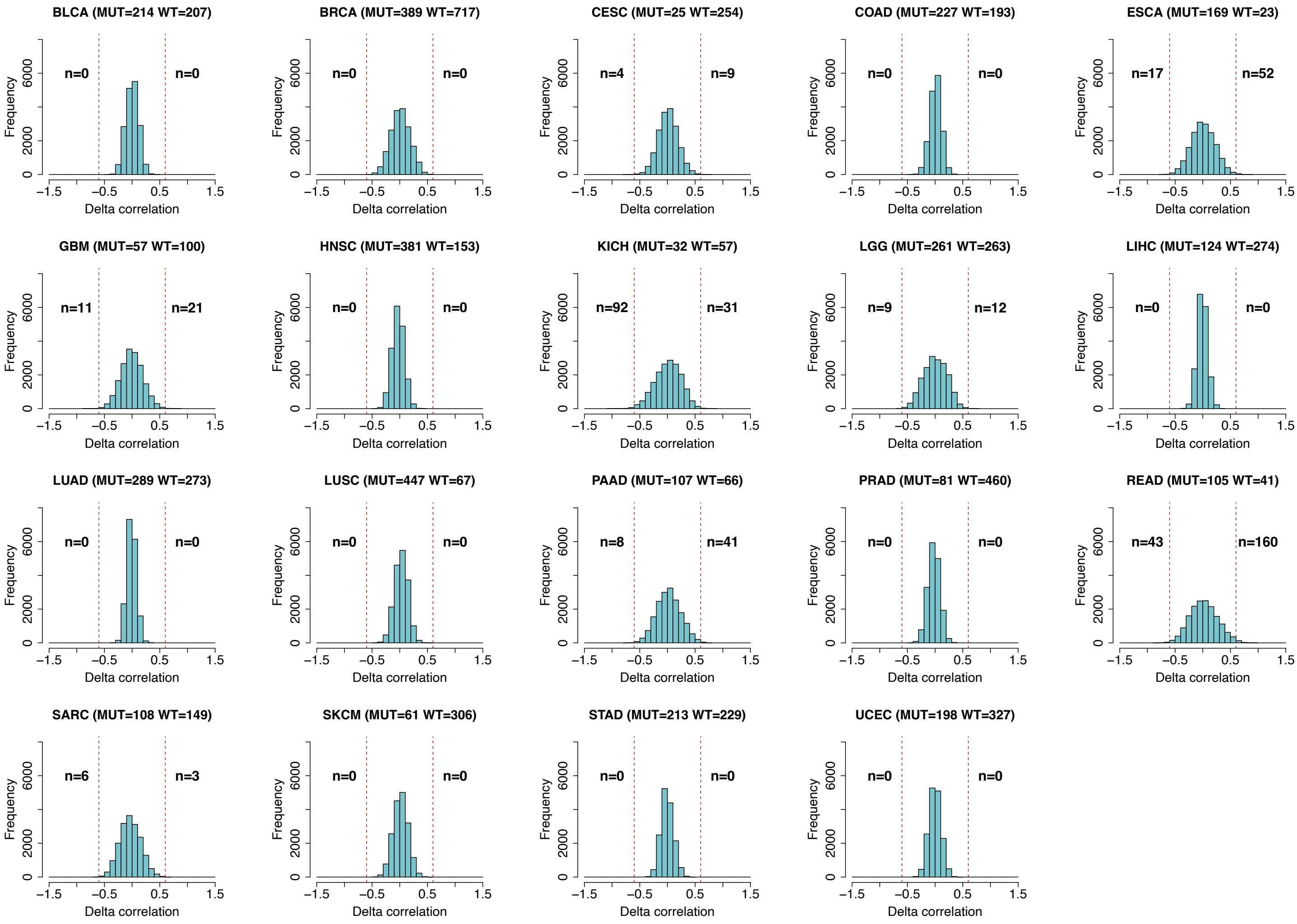

Supplementary figure 6

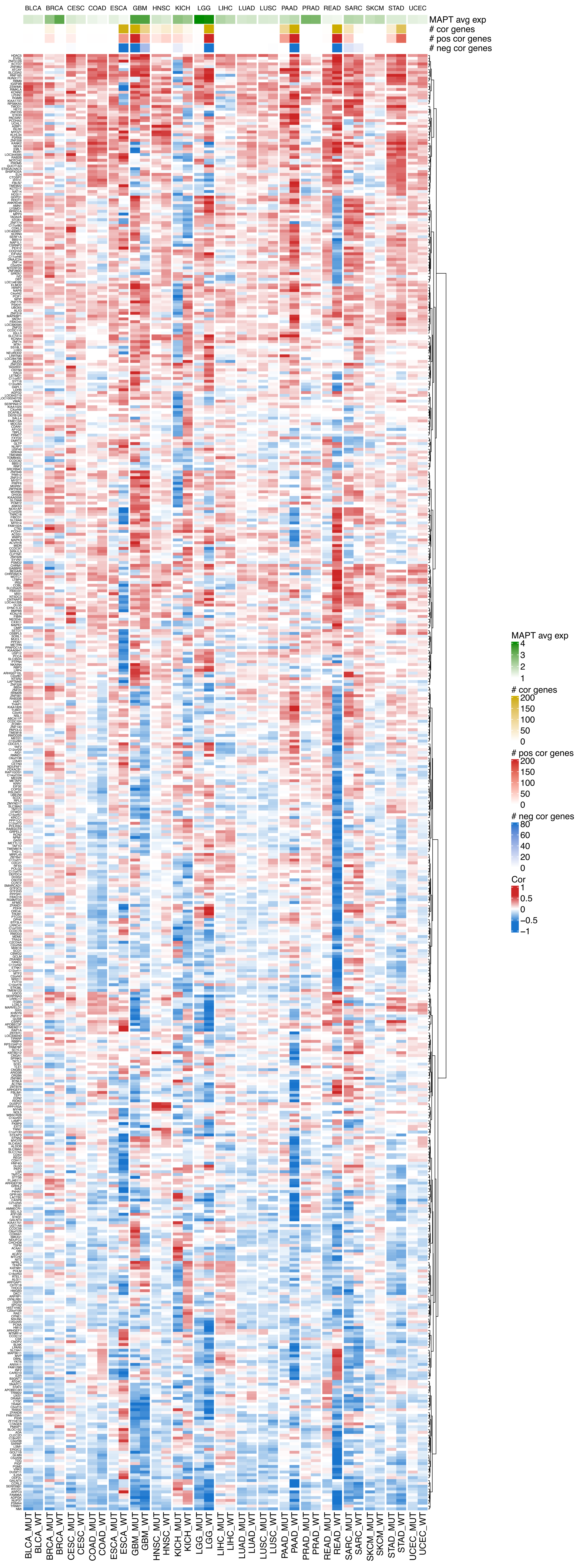

Text

Supplementary figure 7

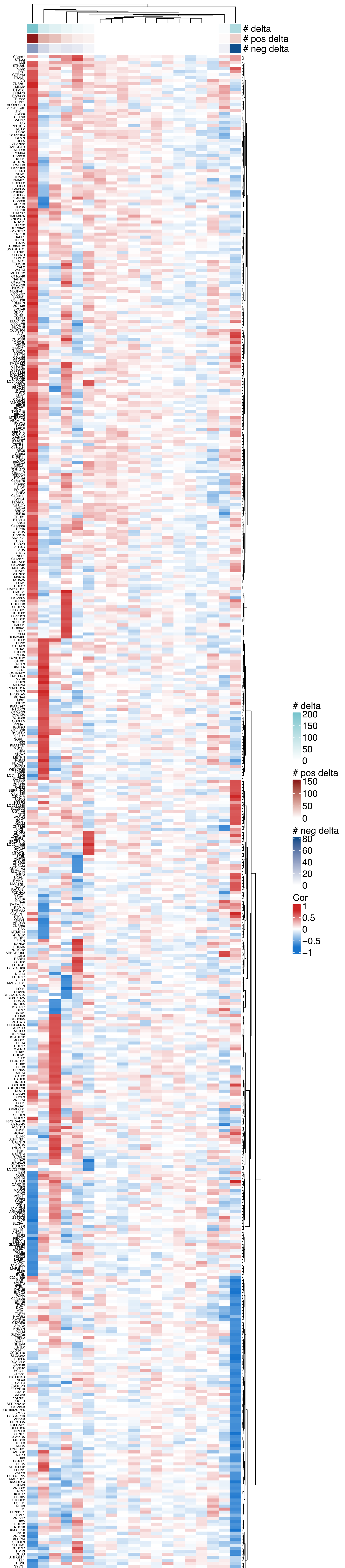

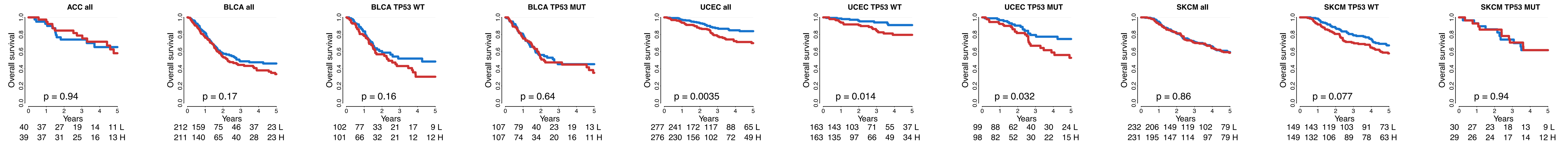

Supplementary figure 8

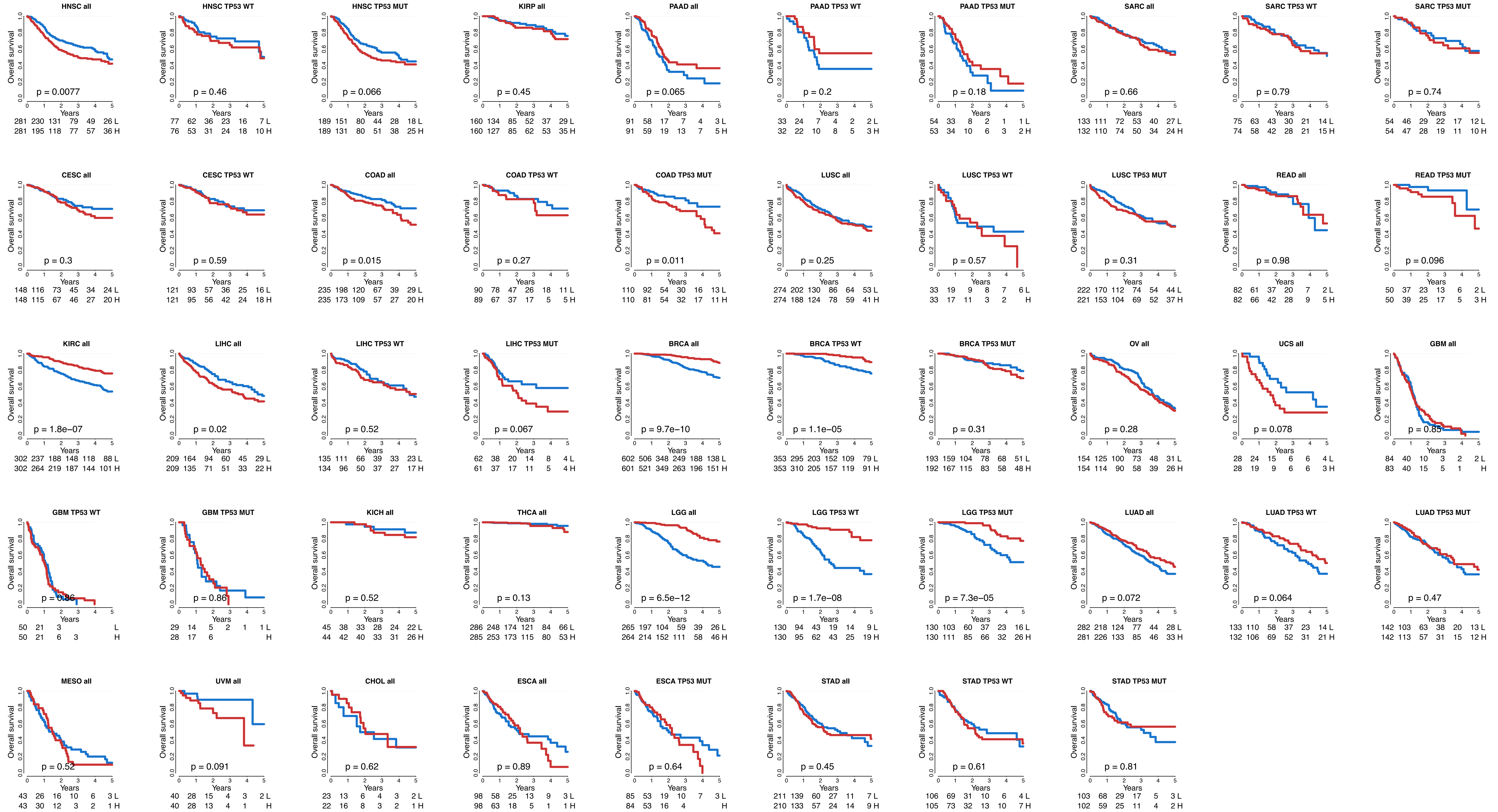

Supplementary Figure 9

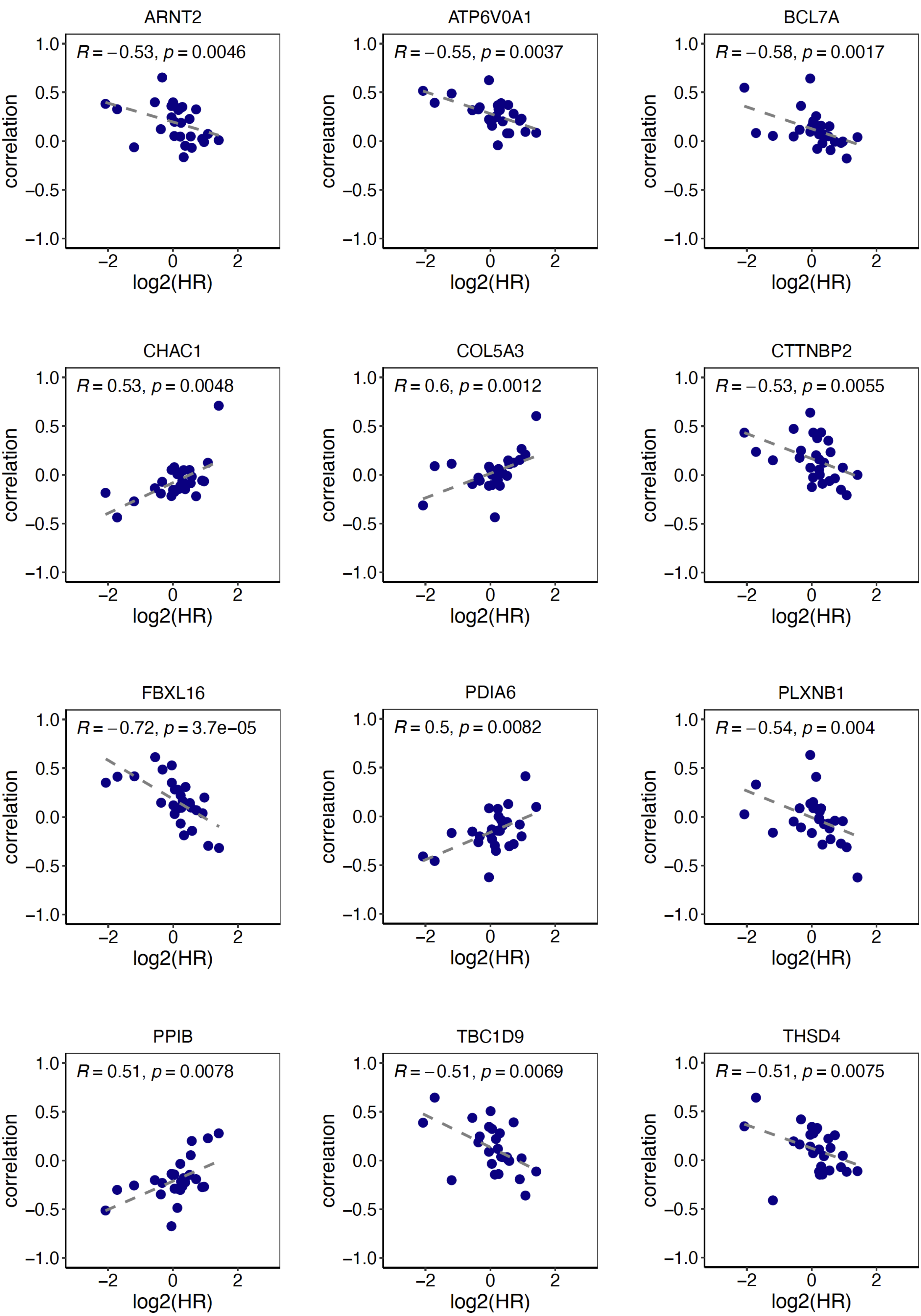

Figure S1 - (a) Correlation of *MAPT* gene expression with all expressed genes. Each histogram shows the correlation value distribution obtained for each cancer type. The number of genes with high positive/negative correlation (i.e. above 0.6 or below -0.6) is reported. (b) For the same analysis in (a), Benjamini-Hochberg adjusted p-values were computed and shown alongside correlation values in volcanoplots. An adjusted p-value threshold of 0.05 is indicated. **(c)** Association between *MAPT* average expression and the number of correlated genes per cancer type. The total number (left), the number of positively correlated genes (centre), and the number of negatively correlated genes (right) were evaluated. **(d)** All cancer types were clustered based on gene correlation with *MAPT*. The ConsensusClusterPlus algorithm was used. The plot shows changes in the 'area under the CDF curve' when increasing the number of clusters 'k', used as a parameter to select the optimal number of clusters, in this case 5. **(e)** Top 20 enriched Hallmark genesets in the comparison between *MAPT* KO and WT neuroblastoma cells.

Figure S2 - Heatmap of 809 genes correlated with *MAPT* expression in at least one cancer type. Both genes and cancer types are ordered based on the hierarchical clustering of the correlation values. Fifty-two of the top 100 genes co-expressed with *MAPT* according to EnrichR (ARCHS^4 dataset) are overlapping with the 809 genes and are annotated in the heatmap. For each cancer type, the average *MAPT* expression and the number of genes correlated with *MAPT* are shown. Cancer types were grouped in 5 distinct clusters based on the correlation profiles using ConsensusClusterPlus and labels are indicated.

Figure S3 – Selected PANTHER pathways in selected cancer types overlaid with the *MAPT*-gene correlation values for all evaluated genes.

Figure S4 - (a) Expression of *MAPT* in *TP53* WT and *TP53* MUT tumors. The analysis is stratified by cancer type. Differences are evaluated by a two-sided Student's t-test. (b) Expression of *MAPT* in tumors with WT P53 or with either a Truncating/HomDel mutation or an Inframe/Missense mutation in PT53. Differences were evaluated by a two-sided Student's t-test.

Figure S5 - Correlation with *MAPT* for all genes was computed separately for TP53 MUT and TP53 WT tumors. The delta correlation distribution (correlation in TP53 MUT – correlation in TP53 WT) is reported in the figure for each cancer type. The number of genes with an absolute delta above 0.6 is indicated.

Figure S6 - Heatmap of 514 genes with an absolute delta correlation above 0.6 in P53 WT vs P53 MUT tumors for at least one cancer type. The correlation analysis was performed only for 19 cancer types with at least 20 patients with P53 WT and 20 P53 MUT. *MAPT* correlation values are shown. For each group identified by the combination of cancer type and P53 status, the average *MAPT* expression and the number of genes correlated with *MAPT* are shown.

Figure S7 - Heatmap of 514 genes with an absolute delta correlation above 0.6 in P53 WT vs P53 MUT tumors for at least one cancer type. The correlation analysis was performed for 19 cancer types with at least 20 patients with P53 WT and 20 P53 MUT.

Figure S8 - *MAPT* cancer-specific association with survival. Kaplan-Meier curves showing the association between *MAPT* (High/Low using the median as threshold) and survival in the overall population and stratifying according to P53 status. P-value obtained by log-rank test

Figure S9 - Scatterplots for genes showing an absolute correlation >0.5 between the *MAPT*-gene correlation and *MAPT* hazard ratio across different cancer types. The 12 genes achieving an absolute *MAPT*-gene correlation >0.6 (as in **Figure 1b**) are shown.

Table S1 Percentage of *TP53* mutations per cancer type. Mutation frequency is separated according to the different mutation types (inframe/missense or truncating/homdel).

Cancer	Inframe/Missense	Truncating/HomDel	WT
ACC	5 (6.58%)	11 (14.47%)	60 (78.95%)
BLCA	136 (32.3%)	78 (18.53%)	207 (49.17%)
BRCA	237 (21.43%)	152 (13.74%)	717 (64.83%)
CESC	15 (5.38%)	10 (3.58%)	254 (91.04%)
CHOL	4 (8.89%)	1 (2.22%)	40 (88.89%)
COAD	160 (38.1%)	67 (15.95%)	193 (45.95%)
DLBC	2 (5.41%)	3 (8.11%)	32 (86.49%)
ESCA	102 (53.12%)	67 (34.9%)	23 (11.98%)
GBM	44 (28.03%)	13 (8.28%)	100 (63.69%)
HNSC	197 (36.89%)	184 (34.46%)	153 (28.65%)
KICH	19 (21.35%)	13 (14.61%)	57 (64.04%)
KIRC	8 (1.91%)	5 (1.19%)	406 (96.9%)
KIRP	6 (1.95%)	2 (0.65%)	299 (97.39%)
LGG	199 (37.98%)	62 (11.83%)	263 (50.19%)
LIHC	72 (18.09%)	52 (13.07%)	274 (68.84%)
LUAD	169 (30.07%)	120 (21.35%)	273 (48.58%)
LUSC	284 (55.25%)	163 (31.71%)	67 (13.04%)
MESO	10 (12.2%)	4 (4.88%)	68 (82.93%)
OV	113 (55.39%)	80 (39.22%)	11 (5.39%)
PAAD	67 (38.73%)	40 (23.12%)	66 (38.15%)
PCPG	1 (0.59%)	2 (1.18%)	166 (98.22%)
PRAD	41 (7.58%)	40 (7.39%)	460 (85.03%)
READ	70 (47.95%)	35 (23.97%)	41 (28.08%)
SARC	51 (19.84%)	57 (22.18%)	149 (57.98%)
SKCM	31 (8.45%)	30 (8.17%)	306 (83.38%)
STAD	130 (29.41%)	83 (18.78%)	229 (51.81%)
TGCT	1 (0.75%)	0 (0%)	132 (99.25%)
THCA	0 (0%)	3 (0.55%)	540 (99.45%)
THYM	3 (2.48%)	2 (1.65%)	116 (95.87%)
UCEC	145 (27.62%)	53 (10.1%)	327 (62.29%)
UCS	39 (69.64%)	12 (21.43%)	5 (8.93%)
UVM	0 (0%)	0 (0%)	80 (100%)

Table S2 Breakdown of pan-cancer TP53 mutational status describing the number of cases for each available classification (based on mutational and copy number status). The table also describe our grouping into WT, inframe/missense or truncating/homdel categories.

Status (mutation;CNA)	count	WT/MUT classification	MUT group
wt;wt	6097	WT	WT
wt;homdel_rec	79	MUT	Truncating/HomDel
Inframe Mutation (putative driver);wt	80	MUT	Inframe/Missense
Inframe Mutation (putative driver);homdel_rec	1	MUT	Truncating/HomDel
Inframe Mutation (putative passenger);wt	1	WT	WT
Missense Mutation (putative driver);wt	2274	MUT	Inframe/Missense
Missense Mutation (putative driver);Amplification	7	MUT	Inframe/Missense
Missense Mutation (putative driver);homdel_rec	31	MUT	Truncating/HomDel
Missense Mutation (putative passenger);wt	16	WT	WT
Truncating mutation (putative driver);wt	1320	MUT	Truncating/HomDel
Truncating mutation (putative driver);Amplification	5	MUT	Truncating/HomDel
Truncating mutation (putative driver);homdel_rec	8	MUT	Truncating/HomDel
